# Bortezomib-based treatment for multiple myeloma patients with renal impairment: A systematic review and meta-analysis of observational studies

**DOI:** 10.1097/MD.0000000000005202

**Published:** 2016-11-18

**Authors:** Wanqiu Zhu, Wenming Chen

**Affiliations:** a Department of Hematology; b Multiple Myeloma Research Center of Beijing, Beijing Chao-Yang Hospital, Capital Medical University, Beijing, People's Republic of China.

**Keywords:** bortezomib, meta-analysis, multiple myeloma, renal insufficiency, systematic review

## Abstract

**Background:**

Renal insufficiency is a common and severe complication of patients with multiple myeloma. The aim of this study was to evaluate bortezomib-based treatment for multiple myeloma patients with renal insufficiency.

**Methods:**

The Cochrane Library, Embase, PubMed, ISI, China National Knowledge Infrastructure, Chinese Biomedical Literature Service System, Chongqing VIP Database, and Wan Fang Data were systematically searched to identify observational studies from January 1, 2001, to December 31, 2015. Myeloma response rate and renal remission rate were pooled by using risk ratio and 95% confidence interval (CI). The Cochran Q and I statistics were used to assess heterogeneity. Sensitivity analysis was performed to test the feasibility of pooled results. Publication bias was conducted when included studies were ≥9. Furthermore, grades of evidence were performed to evaluate study quality.

**Results:**

Eleven retrospective cohort studies were included in the final analysis. The number of available studies and risk ratios (95% CI) were, respectively, 10 and 1.48 (95% CI: 1.28–1.71) for myeloma overall response, 6 and 3.69 (95% CI: 2.22–6.13) for myeloma complete response, 9 and 1.47 (95% CI: 1.28–1.69) for renal overall remission, and 8 and 1.49 (95% CI: 1.26–1.75) for renal complete remission. No significant publication bias was observed and sensitivity analysis confirmed the stability of results. The overall qualities of evidence were high for myeloma complete response and medium for the other 3 outcomes based on the Grading of Recommendations, Assessment, Development and Evaluation system.

**Conclusion:**

Current evidence indicated that bortezomib-based treatment could improve myeloma overall response (especially myeloma complete response) and renal overall remission (including renal complete remission).

## 1 Introduction

Multiple myeloma (MM) is a hematological malignancy characterized by a neoplastic proliferation of plasma cells in the bone marrow, mostly associated with the production of excessive monoclonal immunoglobulin (namely, M protein), which can be either its subclass (e.g., IgG, IgA, IgD, IgE, and IgM) or its light chain (e.g., kappa and lambda) in serum or urine. Evidence exists that median survival of MM is 3 to 4 years with conventional treatment and can be extended to 5 to 7 years with novel agents.

Renal insufficiency is a severe complication of patients with MM that needs to be handled timely. It occurs in 20% to 40% of newly diagnosed patients and a similar percentage of patients develop renal failure during the course of disease.^[1]^ MM patients with renal impairment have a higher mortality and shorter survival time.^[2,3]^ It is reported that the median survival of patients with renal failure was 19.5 versus 40.4 months for patients without renal failure (*P* < 0.001).^[2]^ The median survival of MM patients with severe acute renal injury was only 10 months.^[3]^

Bortezomib is a potent, selective, and reversible inhibitor of the 26S proteasome. In recent years, bortezomib-based regimens have shown activity in 35% to 60% of patients with refractory/relapsed myeloma and in up to 90% of newly diagnosed patients.^[1]^ Researchers have observed that bortezomib-based regimens could improve renal failure and even reverse it.^[1,4,5]^ When corrected ultimately by appropriate treatment, renal failure had no impact on survival.^[6–8]^

However, there are still limited data concerning the reversibility of renal failure and its impacts on survival and safety. To this end, we aimed to synthesize a systematic review and meta-analysis examining the efficacy of bortezomib-based treatment for MM patients with renal insufficiency so as to provide a comprehensive, parsimonious summary of the current evidence on this field.

## 2 Materials and methods

Ethical approval and patient consent were not required for this type of study. The systematic review and meta-analyses were conducted and reported in accordance with the Preferred Reporting Items for Systematic Reviews and Meta-Analyses guidelines.^[9]^ The protocol of this review has been registered in the PROSPERO at www.crd.york.ac.uk/PROSPERO (registration no. CRD42016033961).

### 2.1 Data sources and search strategies

An electronic database at home and that abroad were carefully searched, including the Cochrane Library, Embase, PubMed, ISI (Web of Knowledge), China National Knowledge Infrastructure, Chinese Biomedical Literature Service System, Chongqing VIP Database, and Wan Fang Data. The search strategies were developed using the terms “multiple myeloma” or “plasmacytoma,” “renal” or “kidney,” and “bortezomib” or “velcade” in combination, and adjusted according to the certain database. The search time was run from January 1, 2001, to December 31, 2015. For a detailed search, conference abstracts of American Society of Hematology and American Society of Clinical Oncology, references of the included studies, and relevant supplements were manually searched. In order to identify unpublished or ongoing studies, we grouped according to the ClinicalTrials.gov (www.clinicaltrials.gov/) and International Clinical Trials Registry Platform (http://apps.who.int/trialsearch/). Two investigators independently performed the database search and agreed on final study selection. The detailed search strategies are listed in Table 1.

**Table 1:**
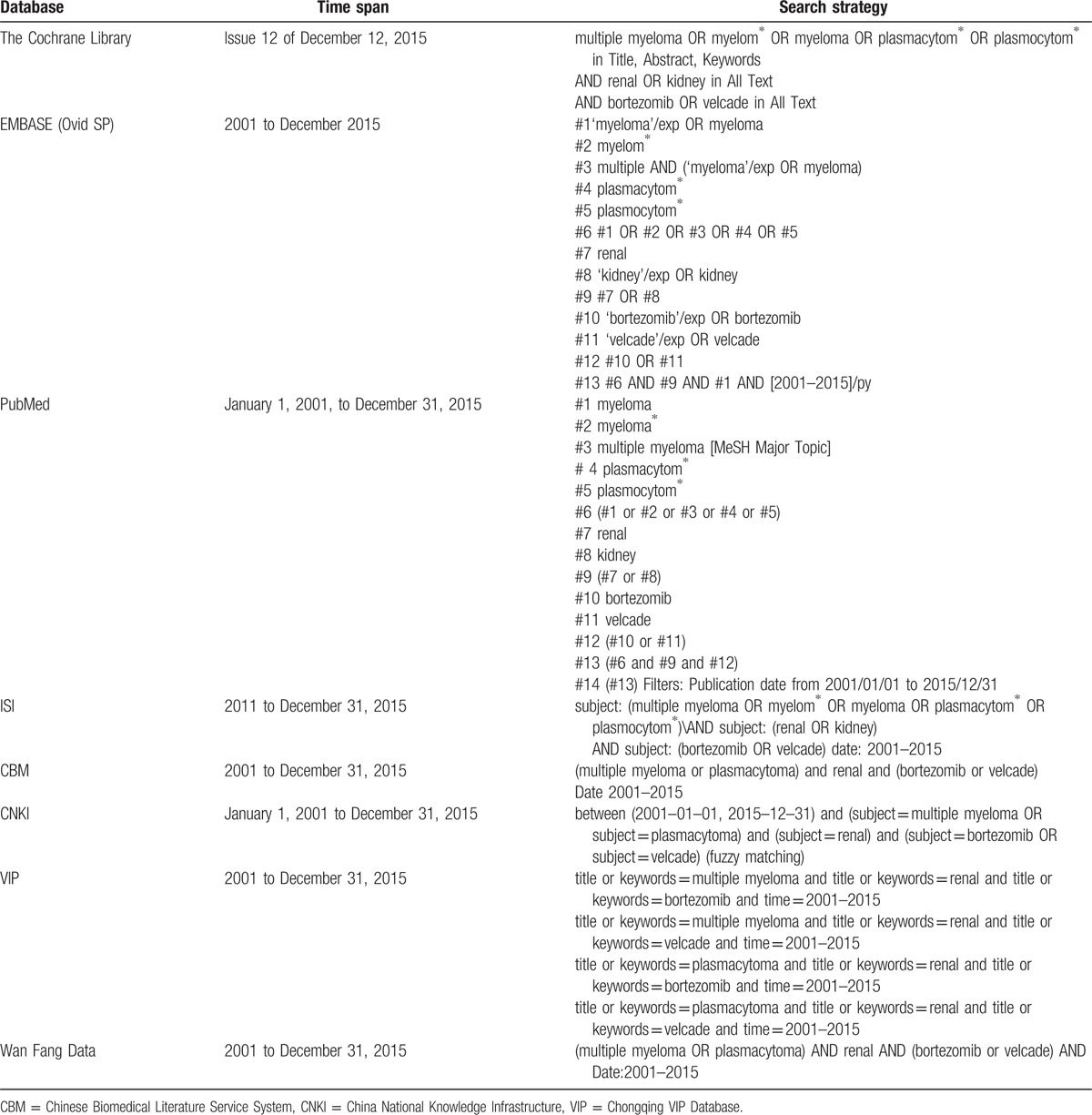
Database search strategies.

### 2.2 Selection criteria

Observational studies comparing bortezomib-containing with non–bortezomib-containing regimens for MM patients with renal insufficiency were selected for meta-analysis if they reported at least 1 of our specified outcomes as myeloma response rate or renal remission rate. Due to personal restrictions, only studies published in English and Chinese were included. For studies that had multiple publications, the publications with longest follow-up or more participants were reserved for extracting data. The eligibility of each study was assessed separately by 2 investigators and the screening results were cross-checked. If a contradiction arose, agreement was achieved through discussion.

### 2.3 Outcomes

Our primary outcomes for this meta-analysis were myeloma overall response (including myeloma complete response) and renal overall remission (including renal complete remission). Secondary outcomes were median progression-free survival, median overall survival, and adverse effects, especially grade 3 or 4 toxicities.

### 2.4 Data extraction

Two reviewers independently extracted information from the selected studies and then double checked with each other. The following data were carefully extracted from relevant studies: publication details (including author and year of publication), study design (cohort study or case–control study), characteristics of population (including setting, study period, size, age, gender), grouping related information (including number and treatment regimens for each group), and outcomes (including curative effects, survival-related data, and adverse effects). When the data required for the analysis could not be extracted, attempts were made to contact the investigators who did the studies.

### 2.5 Evaluation of study quality

The quality of each study was evaluated using a well-established tool, the Newcastle–Ottawa quality scale as recommended by the Cochrane Non-Randomized Studies Methods Working Group.^[10]^ Three main criteria were assessed, including participant selection and representativeness, comparability of study groups, and assessment of outcome or exposure. The score of quality was based on a “star” system (range from 0 to 9 stars)^[11]^; the percentage of the maximum score achieved was used to present the quality of each study. A higher score represented better methodological quality. A high-quality study was defined as a study of ≥7 stars. The reviewers assessed independently and disagreements were resolved by consensus.

### 2.6 Statistical analysis

Statistical analysis was pooled using Review Manager 5.3 software developed by the Cochrane Collaboration. Dichotomous data for cohort studies and case–control studies were expressed as risk ratio (RR) and odds ratio (OR) with 95% confidence intervals (CIs) using the Cochran Mantel–Haenszel method, respectively. RR or OR >1.0 indicated the presence of association between the predictor factor and the outcome considered. Time to event data were pooled and reported as hazard ratio and 95% CIs using the exp[(O − E/V)] method. Heterogeneity was qualitatively assessed by χ^2^ test and quantitatively assessed by the inconsistency index (I^2^). *P* ≥ 0.10 and I^2^ < 50% were deemed to be of no significant heterogeneity. If I^2^ < 25%, the meta-analysis was conducted by fixed-effects model. Otherwise (25% ≤ I^2^ < 50%), random-effects model was used. If I^2^ ≥ 50%, the assumption of homogeneity was deemed invalid and random-effects model was adopted after exploring the causes of heterogeneity by subgroup analysis. Publication bias was assessed visually using a funnel plot based on Begg and Egger method, and was performed only in outcomes consisting of ≥9 studies. Sensitivity analysis was conducted by using the method of leave-1-out, alternative effect measures (RR vs OR), as well as consideration of heterogeneity (random effects vs fixed effects) to test the feasibility of the pooled results.

### 2.7 Overall quality of the evidence

The quality of evidence for the main outcomes was evaluated according to the Grading of Recommendations, Assessment, Development and Evaluation (GRADE) Working Group recommendation with the magnitude of effect, the influence of all plausible residual confounding, and dose–response gradient taken into account.^[12]^ The level of the evidence from observational studies (including cohort study and case–control study) would be upgraded if there were large effects of the intervention/exposure according to the pooling results and dose–response gradient or potential uncontrolled confounding bias might weaken the true effect of the intervention/exposure. We applied the following definitions of quality of the evidence: “high quality,” “moderate quality,” “low quality,” and “very low quality.” Grades of evidence were performed using GRADE profile 3.6. Any discrepancies between the 2 investigators were solved by mutual discussion.

## 3 Results

### 3.1 Study selection

**Figure 1:**
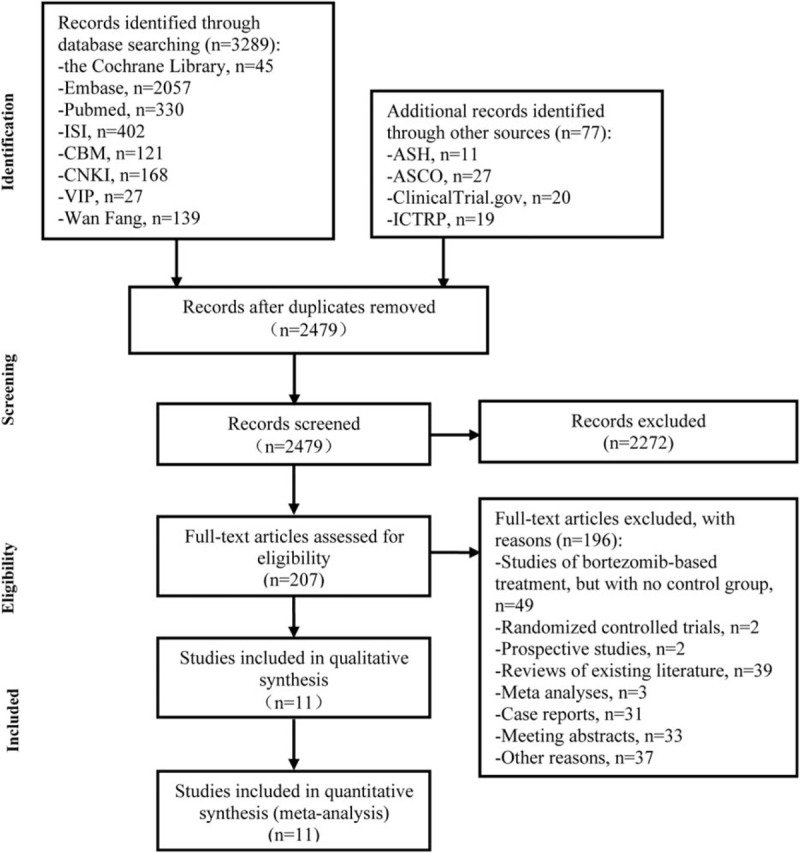
Flow diagram of study selection process. ASCO = American Society of Clinical Oncology, ASH = American Society of Hematology, CBM = Chinese Biomedical Literature Service System, CNKI = China National Knowledge Infrastructure, ICTRP = International Clinical Trials Registry Platform, ISI = Web of Knowledge, VIP = Chongqing VIP Database.

Our initial search yielded 3366 potentially relevant references, of which 887 references were duplicated and 2272 references were deemed ineligible after screening titles and abstracts. Reading the full text of the remaining 207 references led to the exclusion of 196 references. Forty-nine references were excluded because they were evaluating bortezomib treatment but with no control group. Two studies were excluded because they were randomized controlled trials. Two studies were excluded because they were prospective studies. Thirty-nine studies were excluded because they were reviews of the existing literature. Three studies were excluded because they were meta-analyses. Thirty-one studies were excluded because they were case reports. Thirty-three studies were excluded because they were meeting abstracts. Thirty-seven studies were excluded for other reasons. In the end, 11 retrospective cohort studies fully met our inclusion criteria. Of these, 7 studies were published in English^[13–19]^ and 4 studies were published in Chinese.^[20–23]^Figure 1 outlines the flow diagram following Preferred Reporting Items for Systematic Reviews and Meta-Analyses template.

### 3.2 Description of included studies

The 11 retrospective cohort studies contained 961 participants of whom 953 participants were available for analysis; of these, 413 participants received bortezomib-based and 540 participants received non–bortezomib-based treatment. There were 10 studies^[13–20,22,23]^ and 6 studies^[13,14,19,20,22,23]^ available for myeloma overall response (≥partial response) and myeloma complete response. There were 9 studies^[14–21,23]^ and 8 studies^[14–17,19–21,23]^ available for renal overall remission (≥partial response) and renal complete response. The characteristics of the eligible studies are described in Table 2.

**Table 2:**
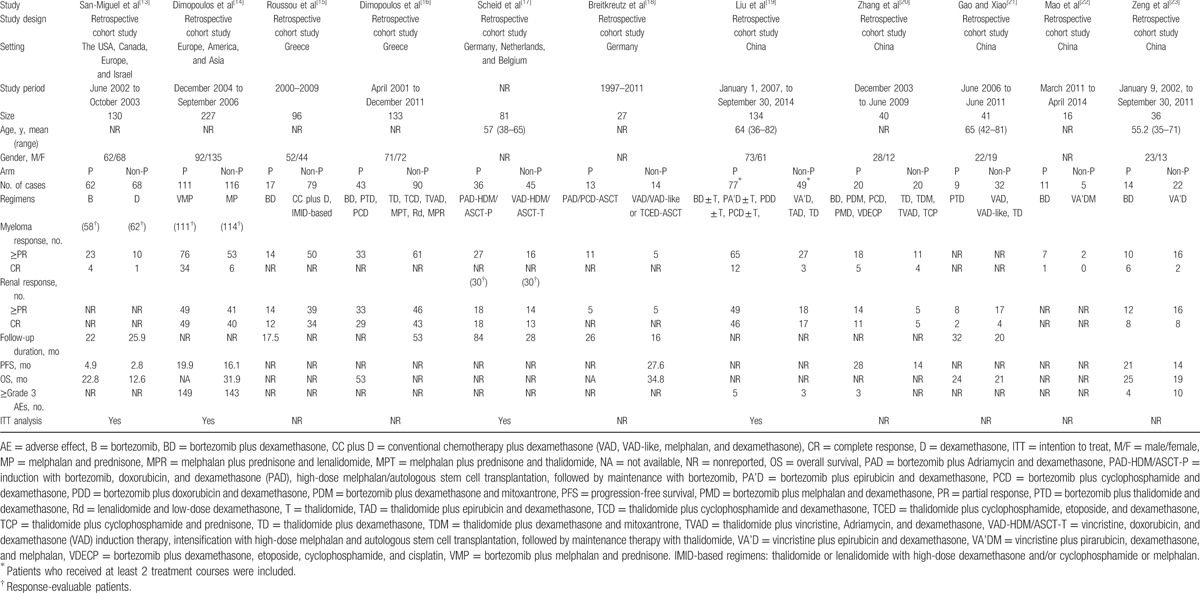
Characteristics of included studies.

### 3.3 Evaluation of study quality

All the studies included had a Newcastle–Ottawa Scale total score of >7 stars, which were “high-quality” studies. Among them, only 1 study^[13]^ scored 9 stars, and the other 10 studies^[14–23]^ scored 7 to 8 stars of which 7 studies^[15,16,18–21,23]^ did not specify whether there was specific control for a second important factor and 1 study^[22]^ did not group based on the way of treatment, so the comparability of them obtained only 1 star. Meanwhile, 6 studies^[14,15,17–19,22]^ did not follow up long enough so that the outcome of them obtained only 2 stars. The detailed progress is shown in Table 3.

**Table 3:**
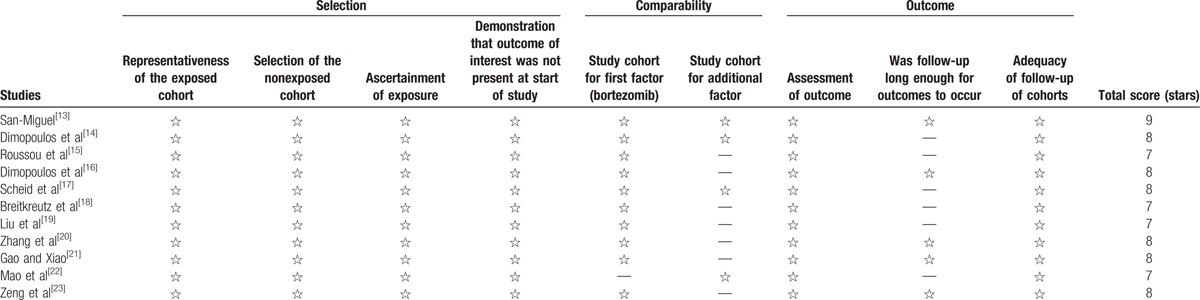
Indices of methodological quality of the studies according to the Newcastle–Ottawa Quality Assessment Scale for cohort studies.

### 3.4 Effects of statistical analysis

#### 3.4.1 Primary outcomes

##### 3.4.1.1 Myeloma response

**Figure 2:**
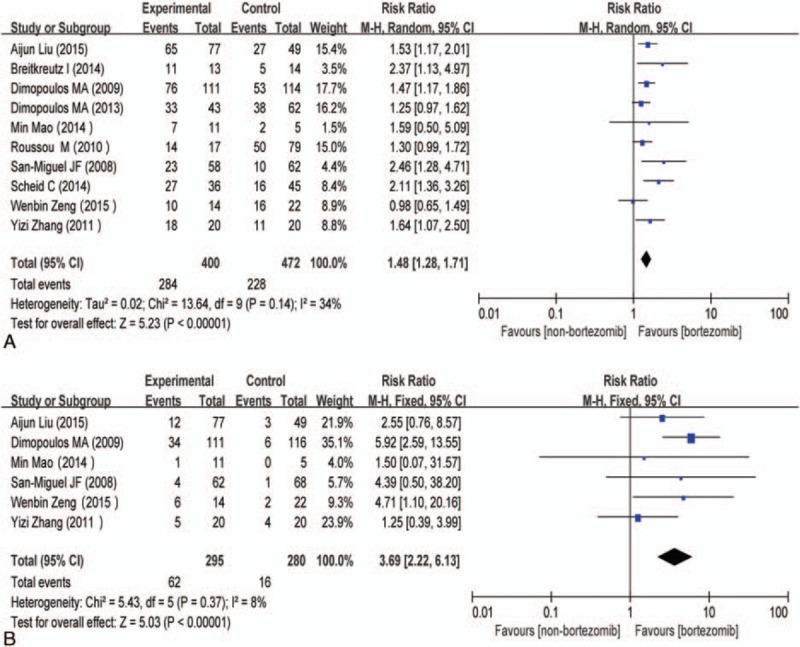
(A) Forest plot of myeloma overall response with bortezomib-based versus non–bortezomib-based treatment for MM patients with renal insufficiency. (B) Forest plot of myeloma complete response with bortezomib-based versus non–bortezomib-based treatment for MM patients with renal insufficiency. CI = confidence interval, control = non–bortezomib-based treatment, experimental = bortezomib-based treatment, MM = multiple myeloma.

There were 10 cohort studies^[13–20,22,23]^ available for myeloma overall response containing 872 participants, including 400 participants who received bortezomib-based treatment and 472 participants who received non–bortezomib-based treatment. Since heterogeneity was observed among the 10 studies (χ^2^ = 13.64, *P* = 0.14; I^2^ = 34%), a random-effects model was adopted for synthesis. The difference was significant among bortezomib- and non–bortezomib-based regimens for MM patients with renal insufficiency, RR = 1.48 (95% CI: 1.28–1.71; *P* < 0.00001) (Fig. 2A). As for myeloma complete response, there were 6 studies^[13,14,19,20,22,23]^ available containing 575 participants, including 295 participants who received bortezomib-based treatment and 280 participants who received non–bortezomib-based treatment. Since no great heterogeneity was observed among the 6 studies (χ^2^ = 5.43, *P* = 0.37; I^2^ = 8%), a fixed-effects model was adopted for synthesis. The difference was significant among bortezomib- and non–bortezomib-based regimens for MM patients with renal insufficiency, RR = 3.69 (95% CI: 2.22–6.13; *P* < 0.00001) (Fig. 2B). Thus, bortezomib treatment resulted in 269% increasing benefit concerning myeloma complete response.

##### 3.4.1.2 Renal response

**Figure 3:**
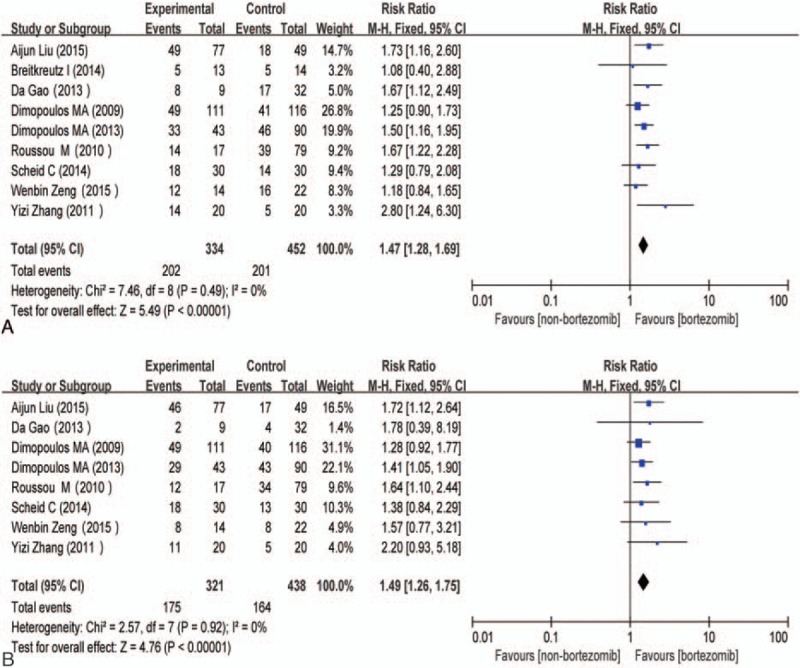
(A) Forest plot of renal overall remission with bortezomib-based versus non–bortezomib-based treatment for MM patients with renal insufficiency. (B) Forest plot of renal complete remission with bortezomib-based versus non–bortezomib-based treatment for MM patients with renal insufficiency. CI = confidence interval, control = non–bortezomib-based treatment, experimental = bortezomib-based treatment, MM = multiple myeloma.

There were 9 studies^[14–21,23]^ available for renal overall remission containing 786 participants, including 334 participants who received bortezomib-based treatment and 452 participants who received non–bortezomib-based treatment. Since no great heterogeneity was observed among the 9 studies (χ^2^ = 7.46, *P* = 0.49; I^2^ = 0%), a fixed-effects model was adopted for synthesis. The difference was significant among bortezomib- and non–bortezomib-based regimens for MM patients with renal insufficiency, RR = 1.47 (95% CI: 1.28–1.69; *P* < 0.00001) (Fig. 3A). There were 8 studies^[14–17,19–21,23]^ available for renal complete response containing 759 participants, including 321 participants who received bortezomib-based treatment and 438 participants who received non–bortezomib-based treatment. Since no great heterogeneity was observed among the 8 studies (χ^2^ = 2.57, *P* = 0.92; I^2^ = 0%), a fixed-effects model was adopted for synthesis. The difference was significant among bortezomib- and non–bortezomib-based regimens for MM patients with renal insufficiency, RR = 1.49 (95% CI: 1.26–1.75; *P* < 0.00001) (Fig. 3B).

#### 3.4.2 Secondary outcomes

Few of the selected studies reported sufficient survival-related data and adverse effects. Thus, the secondary outcomes (including progression-free survival, overall survival, and adverse effects) that we presented upfront could not be analyzed by meta-analysis.

##### 3.4.2.1 Publication bias and sensitivity analysis

**Figure 4:**
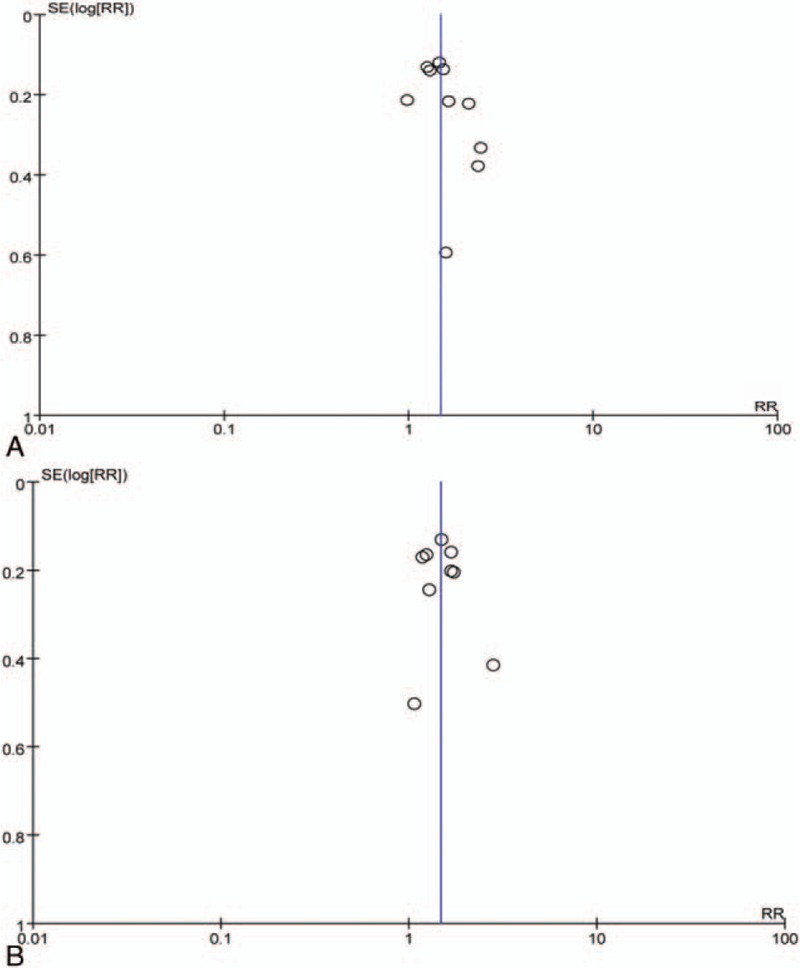
Funnel plot of included studies concerning (A) myeloma overall response and (B) renal overall remission. RR = risk ratio, SE = standard error.

A funnel plot analysis was carried out to detect the publication bias when selected studies were ≥9. The results showed that no significant publication bias was observed (Fig. 4A and B). Sensitivity analysis confirmed the stability of the results. There was no significant change observed concerning the primary outcomes after removing any included study, alternative effect measures (RR vs OR), as well as consideration on heterogeneity (random effects vs fixed effects) (results were omitted).

##### 3.4.2.2 Overall quality of body of evidence

Myeloma complete response was judged to be of high quality in overall quality assessment, which means that further research is very unlikely to change our confidence in the estimate of effect, while the other 3 outcomes were judged to be of moderate quality, which means that further research is likely to have an important impact on our confidence in the estimate of effect and may change the estimate. The evidence summary table based on GRADE system manufactured by GRADE profile 3.6 software is shown in Table 4.

**Table 4:**
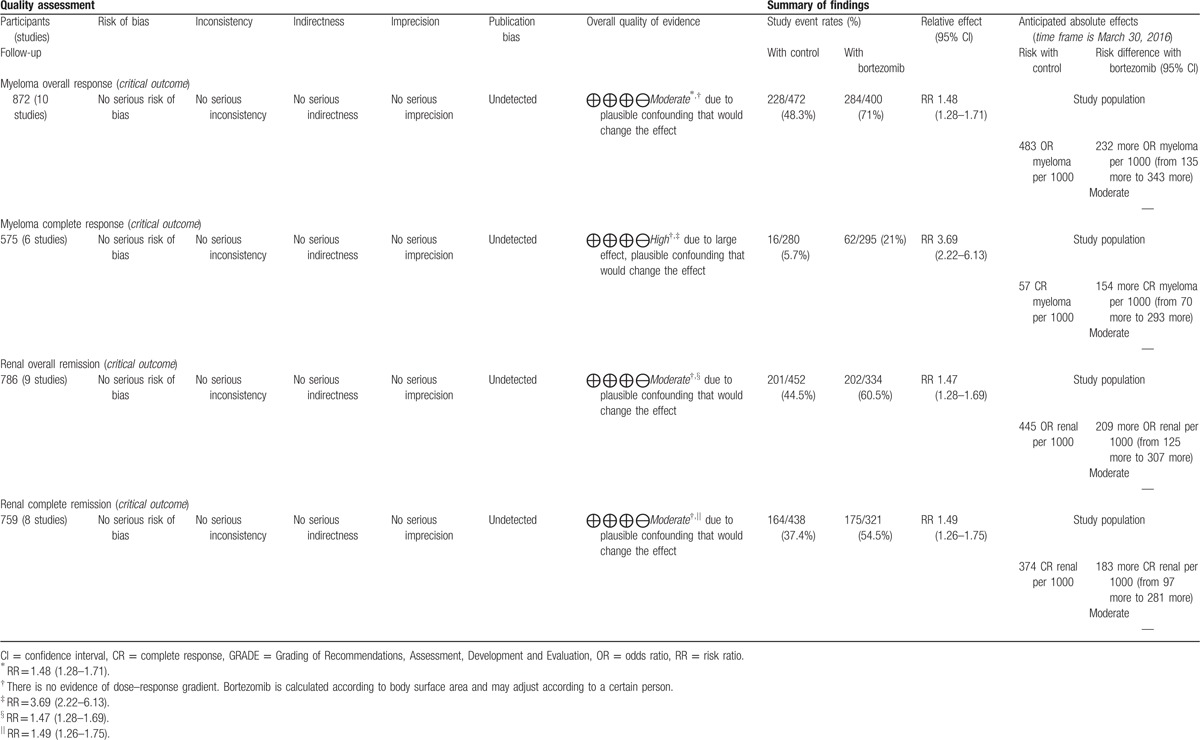
GRADE table.

## 4 Discussion

### 4.1 Summary of evidence

Our systematic review and meta-analysis summarized the efficacy of bortezomib-based treatment for MM patients with renal insufficiency. In our integrated analyses, for MM patients with renal insufficiency, the rates of myeloma overall response (71% vs 48.3%), myeloma complete response (21% vs 5.7%), renal overall remission (60.5% vs 44.5%), and renal complete remission (54.5% vs 37.4%) were higher in bortezomib-based treatment groups than those in non–bortezomib-based treatment groups, indicating that bortezomib-based treatment could improve myeloma overall response and renal overall remission. RRs were 1.48 (95% CI: 1.28–1.71) and 1.47 (95% CI: 1.28–1.69). Notably, a significant benefit was observed for the bortezomib use for MM patients with renal insufficiency on myeloma complete response (RR = 3.69, 95% CI: 2.22–6.13), which showed high quality based on the GRADE system.

### 4.2 Strengths and limitations

Although our meta-analysis strictly followed the recommendation from the Cochrane Collaboration to carry out a comprehensive literature search, statistical analysis, and quality assessment, and adopted the GRADE system to assess the quality of evidence, there were still a number of limitations. First, there were language restrictions. We included only references published in English and Chinese, which might miss some useful data. Second, data of the selected original literatures were uncompleted. There was no thorough report of long-term follow-up, survival, and adverse events, so we could not define the long-term efficacy of bortezomib-based treatment for MM patients with renal insufficiency. Meanwhile, this also affected Newcastle–Ottawa Scale assessment of the study quality. Third, the outcomes were “critical” according to GRADE system, which indicated that the choice of outcome measures was reasonable. But evidence summary based on GRADE system was “moderate” except myeloma complete response, which means that further research is likely to have an important impact on our confidence in the estimate of effect. In addition to myeloma complete response, the RRs of myeloma overall response, renal overall remission, and renal complete remission were all <2; these yield no large or very large and consistent estimates of the magnitude of a treatment effect. Meanwhile, there was no dose–response gradient since the dose of bortezomib was used according to patients’ body surface area and needed to be adjusted whenever necessary. These above-mentioned factors limited upgrading the quality of evidence for the outcomes. As to myeloma complete response, the overall quality of evidence based on GRADE system was high, which means that further research is unlikely to have an important impact on our confidence in the estimate of effect. Thus, the validity is likely to be the same as our estimate (RR = 3.69, 95% CI: 2.22–6.13). Fourth, through our work, we could see that there were some cohort studies regarding bortezomib-based treatment for MM patients with renal insufficiency but the sample sizes were mostly small. In addition, renal insufficiency complicated with MM is often seen as an urgent condition, especially in the case of renal failure. So it is very difficult to carry out randomized controlled trials on this special group of patients, which leads to limitations in this field.

## 5 Conclusions

In conclusion, the finding of the limited present study indicates that bortezomib plays an important role in the treatment of MM patients with renal insufficiency. It can improve myeloma overall response (especially myeloma complete response) and renal overall remission (including renal complete remission). However, due to the small sample size and insufficient data of the included studies, still more studies are needed to further confirm these results.
